# The unexpected finding behind an electrocardiographic abnormality

**DOI:** 10.1093/ehjcr/ytae627

**Published:** 2024-11-25

**Authors:** Beatriz Girela Pérez, Elvira Carrión Rios, Alejandro Gómez Carranza

**Affiliations:** Department of Cardiology, Hospital Universitario Torrecárdenas, Hermandad de Donantes de Sangre s/n, 04009 Almeria, Spain; Department of Cardiology and Image Department, Hospital Universitario Torrecárdenas, Hermandad de Donantes de Sangre s/n, 04009 Almeria, Spain; Critical Care, Hospital Universitario Poniente, Ctra Almerimar, 31, 04700 El Ejido, Almeria, Spain

## Case description

**Figure 1:**
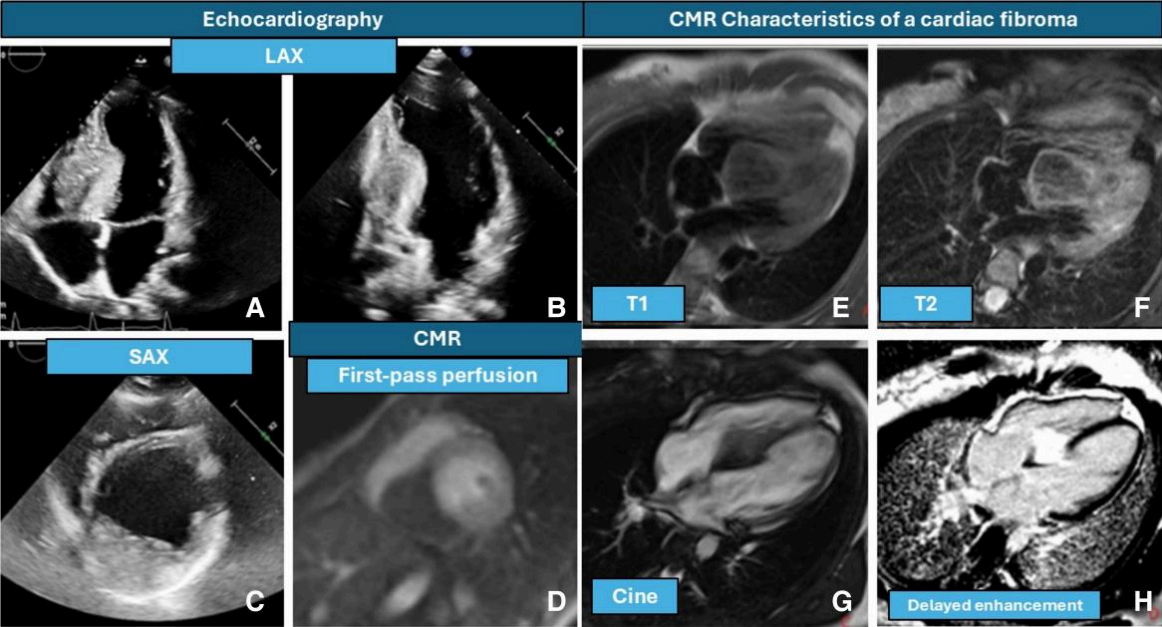
(*A*) Transthoracic echocardiography apical 4C with image compatible with fibroelastoma in the lower septal region. (*B*) Transthoracic echocardiography apical 2C with fibroelastoma in basal inferior segment. (*C*) Short-axis echocardiography, image compatible with fibroelastoma basal inferoseptal. (*D*) First-pass perfusion sequence, basal short-axis plane without initial uptake by the mass. (*E*) T1-weighted spin-echo sequences, hypo-intense/isointense mass with respect to myocardium. (*F*) T2-weighted black blood sequences with hypo-intense/isointense mass compared with healthy myocardium. (*G*) Cine sequences, with image of mass in the lower septum. (*H*) Delayed enhancement study, the mass is uniformly hyper-intense.

A 15-year-old girl, with no family or personal history of interest and asymptomatic, is referred for study due to electrocardiographic abnormalities detected in a sports federation (see Supplementary material online, *Figure S1*). Echocardiography is performed revealing an intra-myocardial mass at the level of the basal inferior septum (*Figure 1A* and *C*) and basal inferior wall (*Figure 1A* and *C*) measuring 48 × 32 mm from the four apical windows and parasternal short axis. No functional repercussion is observed in chambers or valves. The study is completed by magnetic resonance imaging (MRI) showing a mass in the described area, with lobulated borders, without contraction during systole, hypo-isointense T1-weighted (*Figure 1E*), and T2-weighted black blood sequences (*Figure 1F*). In the first-pass perfusion study (*Figure 1D*), contrast is not captured early, and in the delayed enhancement study (*Figure 1H*), the mass is uniformly hyper-intense. The absence of indicators of malignancy (no fat, necrosis, calcifications, or cysts) supports a diagnosis of cardiac fibroma. In the follow-up cardiac MRI after 1 year, there is no change compared with the initial study. Due to the absence of progression in imaging tests, the absence of clinical repercussion, and no malignancy data, close clinical follow-up has been recommended. In cases like this, if the fibroma is small and asymptomatic, a conservative approach with periodic imaging typically every 6–12 months is recommended. If the patient experiences symptoms such as ventricular arrhythmias, syncope, heart failure, chest pain, or haemodynamic compromise, surgical removal should be considered.^1^ Unlike other tumours, fully excised cardiac fibromas generally do not recur, which benefits long-term prognosis. Although uncommon, fibromas are the second most common paediatric cardiac tumour and can also occur in adults. They usually arise in the ventricular myocardium and can grow considerably. Unlike rhabdomyomas, fibromas do not regress spontaneously, and their location is more frequently located in the left ventricle. A high index of suspicion is crucial. First, cardiac fibroma can account for electrocardiographic abnormalities by altering intra-ventricular conduction by compressing or disrupting the conduction tissue in the left ventricle, leading to delayed electrical impulse conduction and electrocardiogram manifestations such as QRS widening and repolarization abnormalities.^2^ Cardiac MRI enables precise delineation of cardiac masses and their relationship with adjacent structures, as well as non-invasive tissue characterization, which is critical for diagnosing and managing cardiac tumours.^3^

## Supplementary Material

ytae627_Supplementary_Data

## Data Availability

The data underlying this article are available in the article.

## Notes

**Consent:** I confirm that I have written consent from the patient to publish their data in compliance with COPE guidelines.

**Funding:** None declared.

## References

[1] Hasnie AA, Muthukumar L, Galazka P, Schmidt L, Khraisat A, Crouch J, et al Large symptomatic ventricular fibromas: a surgical challenge. CASE: Cardiovascular Imaging Case Reports 2023;7:354–359. 37791123 10.1016/j.case.2023.04.007 PMC10542748

[2] Kurmann R, El-Am E, Ahmad A, Abbasi MA, Mazur P, Akiki E, et al Cardiac masses discovered by echocardiogram: what to do next? Structural Heart 2023;7:100154. 37520139 10.1016/j.shj.2022.100154 PMC10382990

[3] Bangolo A, Fwelo P, Iyer KM, Klinger S, Tavares L, Dey S, et al Primary cardiac sarcoma: clinical characteristics and prognostic factors over the past 2 decades. Diseases 2023;11:74. 37218887 10.3390/diseases11020074 PMC10204403

