# Supplementary figures and images for: The unexpected finding behind an electrocardiographic abnormality
Source: Eur Heart J Case Rep. 2024 Nov 25;8(12):ytae627. doi: 10.1093/ehjcr/ytae627 (PMC11630830; doi:10.1093/ehjcr/ytae627)

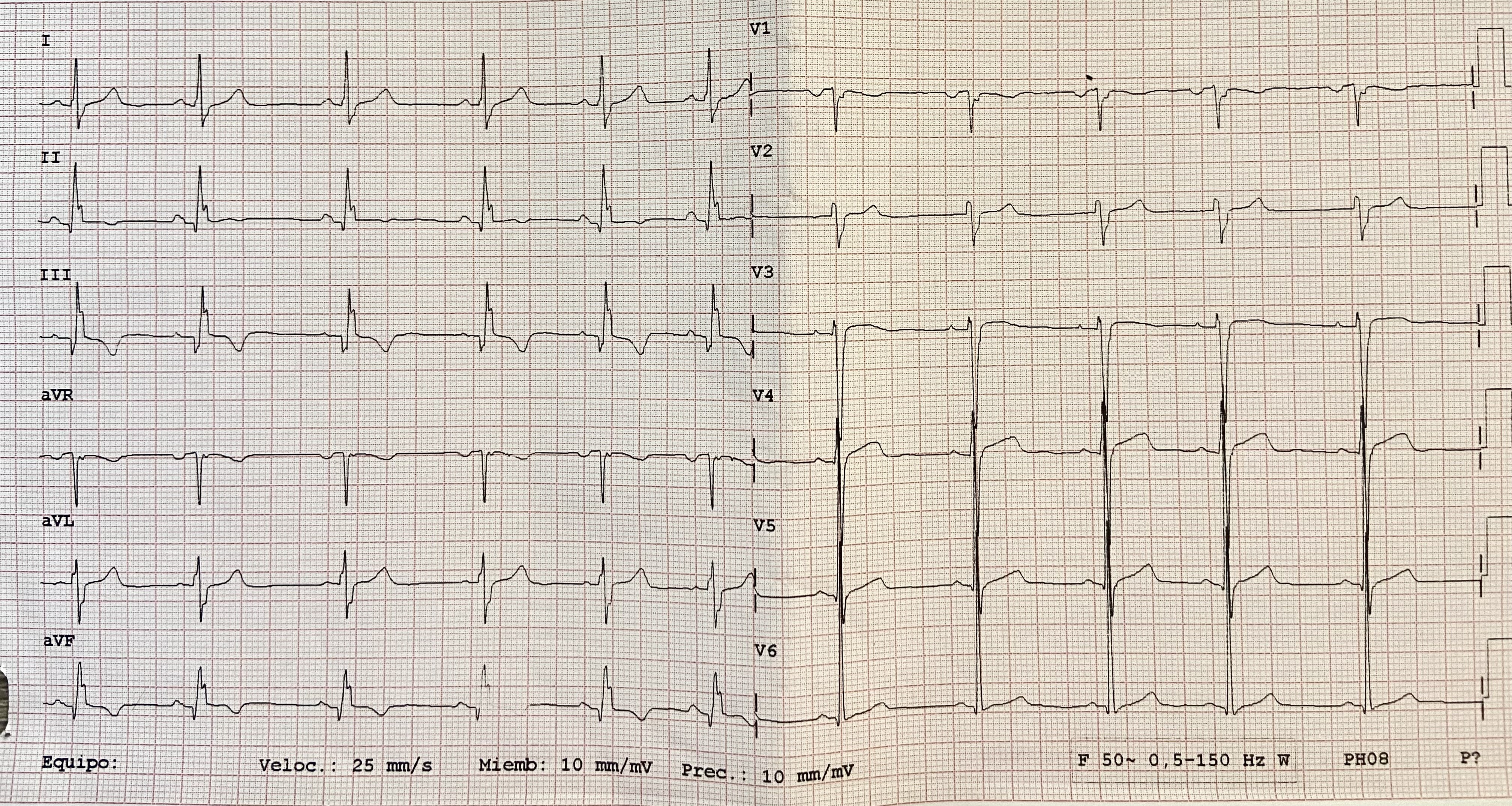

Supplement: ytae627_Supplementary_Data [file ytae627_supplementary_data.jpeg]
